# Maternal motor vehicle crashes during pregnancy and child neurodevelopment

**DOI:** 10.1038/s41390-024-03740-0

**Published:** 2024-11-12

**Authors:** Asma M. Ahmed, Allie Sakowicz

**Affiliations:** 1 Department of Epidemiology and Prevention, Wake Forest University School of Medicine, Winston-Salem, NC USA; 2 Department of Obstetrics and Gynecology, Wake Forest University School of Medicine, Winston-Salem, NC USA; 3 Department of Pediatrics, Wake Forest University School of Medicine, Winston-Salem, NC USA

Maternal motor vehicle crashes (MVCs) during pregnancy represent a significant public health issue, affecting as many as 3% of pregnancies in some US states.^1^ MVCs are the leading cause of injury-related death during pregnancy and fetal mortality, and are associated with a range of adverse maternal and infant outcomes.^2^ Beyond the immediate perinatal complications, there is growing interest in understanding the broader implications of MVCs on offspring, particularly potential links to neurodevelopmental disorders (NDDs) in children. NDDs affect ~8.5% of children under five years old worldwide, representing a major global health concern due to their lifelong impact on individuals and families.^3^ The etiology of NDDs is complex and not fully understood, involving both genetic and environmental factors. While traditionally overlooked, a growing body of literature highlights the role of maternal exposures during pregnancy—such as maternal injuries in general and MVCs in particular—as contributors to the risk of developing NDDs.^4,5^

In this issue of *Pediatric Research*, Chang and colleagues conducted a population-based study in Taiwan to examine the association between maternal MVCs during pregnancy and NDDs in children, including autism spectrum disorder (ASD), intellectual disability (ID), attention-deficit/hyperactivity disorder (ADHD), and infantile cerebral palsy (CP).^6^ The study compared the occurrence of NDDs in offspring exposed *in-utero* to MVCs (*n* = 19,277) with matched controls unexposed to such injuries (*n* = 76,015). The study found modest increases in the risk of ASD, ID, and ADHD after *in-utero* exposure to MVCs. The hazard ratios (HRs), adjusted for maternal demographic and lifestyle factors, were as follows: ID: 1.33 (1.10–1.60); ADHD: 1.14 (1.05–1.23); ASD: 1.21 (1.00–1.45). These associations varied by injury timing and severity, with higher risks for ASD after 3^rd^-trimester injuries and for ID after severe injuries. Additionally, an increased risk of CP was found, but only with severe injuries (maximum abbreviated injury scale (MAIS) > 3, adjusted HR: 3.86 (1.27–11.78)).

Chang’s study used police reports to identify MVCs during pregnancy. These reports offer objective details about the accidents, such as timing, severity, contributing factors, seat belt use, and airbag availability.^1,7^ However, police reports may be influenced by factors like car insurance policies, legal driving age, and the driver’s demographic and socioeconomic characteristics, potentially leading to underreporting of minor accidents. In contrast, administrative health data that include both hospitalized and non-hospitalized injuries may capture a broader range of MVCs injuries but lack specific accident details. Still, clinical data can be affected by patient characteristics and healthcare-seeking behaviors. Notably, Chang’s findings revealed no associations for MVCs that did not result in a clinical visit, suggesting that clinically significant MVCs injuries are typically reflected in clinical data. Ideally, combining police and clinical data would provide a more comprehensive view, reducing both underreporting and misclassification bias.

Administrative data also present challenges due to their limited detail on important confounders, such as individual-level socioeconomic status (SES) indicators like education, income, and occupation. Additionally, maternal morbidities not considered in Chang’s study, such as mental health conditions, may confound these associations, as they are linked to both injury risk during pregnancy and NDDs in offspring. In our ongoing research using electronic health record data from Atrium Health Wake Forest Baptist health system, we found higher rates of maternal mental illness documented prior to pregnancy among those involved in MVCs during pregnancy compared to those who were not (17.2% vs 10.5%; crude risk ratio of 1.63). Similarly, the rates of mental illness diagnosed during pregnancy were 22.3% and 17.8%, respectively (crude risk ratio of 1.25) *(unpublished data)*. Sensitivity analyses for unmeasured confounding, such as quantitative bias analyses or negative controls, may help mitigate these issues in studies relying on administrative data.^8,9^

The occurrence of MVCs is not randomly distributed across the population. Chang’s study found that maternal MVCs were more likely among women living in rural areas and those with low incomes. Social determinants of health—such as SES, geographic location, and neighborhood environment—may play a crucial role in the risk of MVCs during pregnancy and their potential impacts on child neurodevelopment.^1,10^ Women with low SES, low education levels, or those living in rural or underserved areas may have limited access to prenatal care and safety information (e.g., proper seat belt use), face barriers to safe transportation, or live in areas with poorly maintained infrastructure, all of which may increase their risk of car accidents.^11^ Additionally, these women may encounter difficulties in accessing timely and adequate medical attention after an accident, potentially delaying the detection of complications that could affect the developing fetus.^10^ This underscores the need for further research into the disparities surrounding the burden and impact of maternal MVCs.

Disparities in the global burden of MVCs are also evident.^12,13^ Low- and middle-income countries bear a disproportionate burden, despite having fewer vehicles than high-income nations.^13^ While the disability-adjusted life years (DALYs) rates for road injuries in high-income countries declined between 1990 and 2013, some regions, such as South Asia and West and South sub-Saharan Africa, experienced increases over the same period.^12^ Similarly, road traffic deaths have decreased in many high-income countries from 2013 to 2016, but only 23.5% of middle-income countries and none of the low-income countries have achieved such reductions.^13^ These trends emphasize the need for more research in high-burden areas to better understand the impact of MVCs on maternal and child health outcomes.

Although not addressed in Chang’s study, previous research has shown poorer perinatal outcomes among women who do not wear seat belts. For instance, in a population-based study in the state of Utah (1992–1999 births), pregnant women not wearing a seat belt during an MVC were 2.3 times more likely to experience fetal death compared to those who were wearing one.^14^ Seat belts are recommended for all vehicle occupants, and while the majority of pregnant women report using seat belts, only about a third use them correctly.^15^ The American College of Obstetricians and Gynecologists advises counseling on proper seat belt use, but adherence varies, with some states reporting that only about a third of women receive such counseling.^2,16^ Emphasizing the importance of prenatal counseling on proper seat belt use could play a key role in reducing adverse maternal and fetal outcomes following MVCs.

Early identification of NDDs allows for earlier intervention, which may help reduce symptom severity and improve developmental outcomes.^17^ Chang’s study findings may highlight the potential value of enhanced neurodevelopmental screening for children exposed to maternal injuries. Future research could examine the benefits of incorporating regular inquiries about maternal injuries in well-child visits to detect early signs of developmental delays, facilitating timely interventions.

In summary, as evidence continues to accumulate on the negative impact of maternal injuries on maternal and child outcomes, it may be time to reconsider current clinical guidelines, which primarily focus on immediate post-injury monitoring and lack ongoing assessment for longer-term complications. The study by Chang and colleagues contributes to this growing body of research and underscores the need for further investigation to help improve maternal and child outcomes following injuries.
